# Minimization of Childhood Maltreatment Is Common and Consequential: Results from a Large, Multinational Sample Using the Childhood Trauma Questionnaire

**DOI:** 10.1371/journal.pone.0146058

**Published:** 2016-01-27

**Authors:** Kai MacDonald, Michael L. Thomas, Andres F. Sciolla, Beacher Schneider, Katherine Pappas, Gijs Bleijenberg, Martin Bohus, Bradley Bekh, Linda Carpenter, Alan Carr, Udo Dannlowski, Martin Dorahy, Claudia Fahlke, Ricky Finzi-Dottan, Tobi Karu, Arne Gerdner, Heide Glaesmer, Hans Jörgen Grabe, Marianne Heins, Dianna T Kenny, Daeho Kim, Hans Knoop, Jill Lobbestael, Christine Lochner, Grethe Lauritzen, Edle Ravndal, Shelley Riggs, Vedat Sar, Ingo Schäfer, Nicole Schlosser, Melanie L Schwandt, Murray B Stein, Claudia Subic-Wrana, Mark Vogel, Katja Wingenfeld

**Affiliations:** 1 Department of Psychiatry, University of California San Diego, San Diego, California, United States of America; 2 Department of Psychiatry & Behavioral Sciences, University of California Davis, Davis, California, United States of America; 3 Expert Center Chronic Fatigue, Radboud University Medical Center, Nijmegen, Netherlands; 4 Universität Heidelberg, Central Institute of Mental Health, Heidelberg, Germany; 5 Atlanta VA Medical Center, Atlanta, Georgia, United States of America; 6 Department of Psychiatry, Emory University, Atlanta, Georgia, United States of America; 7 Brown University, Providence, Rhode Island, United States of America; 8 University College Dublin, Dublin, Ireland; 9 Department of Psychiatry, University of Münster, Münster, Germany; 10 Department of Psychology, University of Canterbury, Christchurch, New Zealand; 11 Department of Psychology, University of Gothenburg, Gothenburg, Sweden; 12 Bar Ilan University, Ramat Gan, Israel; 13 School of Health Sciences, Jönköping University, Jönköping, Sweden; 14 Department of Medical Psychology and Medical, University of Leipzig, Leipzig, Germany; 15 Department of Psychiatry and Psychotherapy, Helios Hospital Stralsund, Stralsund, Germany; 16 Department of Psychiatry and Psychotherapy, University Medicine Greifswald, Greifswald, Germany; 17 University of Sydney, Sydney, Australia; 18 Department of Psychiatry, Hanyang University Medical School, Seoul, South Korea; 19 Department of Clinical Psychology, Faculty of Psychology and Neuroscience, Maastricht University, Maastricht, Netherlands; 20 University of Stellenbosch, MRC Unit on Anxiety & Stress Disorders, Department of Psychiatry, Stellenbosch, South Africa; 21 Norwegian Institute for Alcohol and Drug research (SIRUS), and Norwegian Centre for Addiction Research (SERAF), University of Oslo, Oslo, Norway; 22 University of North Texas, Denton, Texas, United States of America; 23 Koç University Medical School, Istanbul, Turkey; 24 Department of Psychiatry and Psychotherapy, University Medical Center Hamburg-Eppendorf, Hamburg, Germany; 25 Research Department, Evangelical Hospital Bielefeld, Bielefeld, Germany; 26 National Institute on Alcohol Abuse and Alcoholism, Bethesda, Maryland, United States of America; 27 Department für Psychosomatic Medicine and Psychotherapy, University Medical Center of Mainz University, Mainz, Germany; 28 Division of Substance Use Disorders, Psychiatric Hospital of the University of Basel, Basel, Switzerland; 29 Department of Psychiatry, Charité University Berlin, Campus Benjamin Franklin, Berlin, Germany; The University of Queensland, AUSTRALIA

## Abstract

Childhood maltreatment has diverse, lifelong impact on morbidity and mortality. The Childhood Trauma Questionnaire (CTQ) is one of the most commonly used scales to assess and quantify these experiences and their impact. Curiously, despite very widespread use of the CTQ, scores on its Minimization-Denial (MD) subscale—originally designed to assess a positive response bias—are rarely reported. Hence, little is known about this measure. If response biases are either common or consequential, current practices of ignoring the MD scale deserve revision. Therewith, we designed a study to investigate 3 aspects of minimization, as defined by the CTQ’s MD scale: 1) its prevalence; 2) its latent structure; and finally 3) whether minimization moderates the CTQ’s discriminative validity in terms of distinguishing between psychiatric patients and community volunteers. Archival, item-level CTQ data from 24 multinational samples were combined for a total of 19,652 participants. Analyses indicated: 1) minimization is common; 2) minimization functions as a continuous construct; and 3) high MD scores attenuate the ability of the CTQ to distinguish between psychiatric patients and community volunteers. Overall, results suggest that a minimizing response bias—as detected by the MD subscale—has a small but significant moderating effect on the CTQ’s discriminative validity. Results also may suggest that some prior analyses of maltreatment rates or the effects of early maltreatment that have used the CTQ may have underestimated its incidence and impact. We caution researchers and clinicians about the widespread practice of using the CTQ without the MD or collecting MD data but failing to assess and control for its effects on outcomes or dependent variables.

## Introduction

Childhood maltreatment is both prevalent and impactful [1, 2]. Correlates of these adverse early experiences include increased stress responses [3], dysfunctional regulation of glucocorticoid signaling [4], impaired psychological functioning [5], adult intimate partner violence [6], a variety of mental illnesses [1, 7, 8], suicide attempts and suicides [9, 10], and all cause morbidity and mortality [11, 12]. Due to its ubiquity—as well as its myriad, cumulative effects on the developing mind, brain, body, and relationships—early maltreatment is perhaps the most important general historical factor to assess in a variety of health care contexts [13, 14].

Though more nuanced and sensitive tools for quantifying early maltreatment are in development [15], one of the most commonly-used and well-validated measures—with over 1,000 citations—is the Childhood Trauma Questionnaire (CTQ) ([16], [17] for review). This scale measures five categories of childhood maltreatment: Emotional, Sexual and Physical Abuse (EA, SA and PA), and Emotional and Physical Neglect (EN and PN) (Bernstein & Fink, 1998). Scores on the CTQ, specifically, correlate with both the onset and course of mental illness [1, 18, 19], markers of cellular aging [20], important psychological parameters like stereotype awareness and temperament [21, 22], as well as the structure, function and connectivity of critical brain regions associated with resilience and vulnerability to life stressors (i.e. amygdala) [23–27].

In spite of the fact that evidence suggests moderate to good consistency of self-reports of maltreatment over time [28], the retrospective nature of the CTQ means that response bias has the potential to undermine its validity. Aware of this issue, and that underreporting is a greater risk than over-reporting [29], the CTQ scale’s authors included in it a 3-item response bias subscale called the Minimization-Denial (MD) scale. Attesting to this subscale’s perceived import, the MD scale survived the CTQ’s abridgement from a 70-item scale to its current 28-item version: the most ubiquitous version in current use [30].

In the CTQ manual, the scale’s authors warn that responses of “very often true” to any one of the three MD items may suggest underreporting of childhood trauma (Bernstein & Fink, 1998). Despite this caveat, the overwhelming majority of studies that report CTQ data do not mention the MD items or take them into account in analyses (but see [31] and [25] and [32] for discussion). Arguably, this curious, widespread, systematic omission assumes, *de facto*, either that: 1. the incidence of minimization (i.e. defined herein as a positive MD score) is too rare to warrant examination; or 2. the MD scale does not serve its intended purpose and has no bearing on results. Importantly—as far as we are aware—neither of these assumptions has been systematically examined until recently. Regarding the incidence of minimization: as discussed in a prior publication [32], it is relatively common (10–40% of respondents). Regarding the impact of minimization on reported rates of maltreatment—or on the relationship between maltreatment and outcomes of interest—information is lacking. To address the peculiar vacuum in the literature on the MD scale’s characteristics, frequency, and import, we designed the present study, examining the CTQ and MD scores of a large, varied population of clinical (psychiatric) and community subjects.

Regarding the specific details of the MD scale, items answered “very often true” (hereafter, “MD-positive”) convey a naïvely positive, almost idyllic representation of childhood experiences. These particular items somewhat hyperbolically suggest that: 1) there was “nothing” the person wanted to change about their family; 2) their childhood was “perfect”; and 3) their family was the “best […] in the world” (Bernstein & Fink, 1998). Notably, scoring of the MD scale differs from the regular CTQ items. While the CTQ’s abuse and neglect scales are scored based on sums of polytomous item ratings (range of 1 to 5), MD items are dichotomized: scores of 1 through 4 are coded as 0 and scores of 5 (“very often true”) are coded as 1. This dichotomous coding system is thought to isolate “exaggeratedly desirable responses” [33]. In one of the few examinations of the MD items, Gerdner and Allgulander (2009) suggested that when raw, polytomous responses on these 3 items are summed, a new, nonpathological subscale (which they called Idealization of the Upbringing scale) can be created [31]. They furthermore found that MD—but not Idealization of the Upbringing—is correlated with a social desirability scale, the Marlowe-Crowne Social Desirability Scale [34]. Thus, it is also important to distinguish between dichotomous versus polytomous scoring of MD items, which appear to indicate different constructs.

Originally, the MD scale was validated against the Balanced Inventory of Desirable Responding [35]. Both the self-deception and impression management subscales of the Balanced Inventory of Desirable Responding were strongly and positively correlated with MD, in contrast to their negative correlations with the CTQ’s five primary scales [33]. As mentioned above, subsequent studies like have confirmed that the MD scale correlates with other response bias measures [31]. The real-world consequence of this response bias on the CTQ, however, is understudied. That is, even if we accept that the MD scale indicates a social desirability bias, it is still not clear 1) whether this bias has a positive or negative connotation; or 2) whether such a bias has a meaningful impact on the validity of the CTQ and its role in clinical research and practice.

Importantly, context influences response biases. For example, bias indicators are both common and well-studied in forensic settings [36]. In terms of the CTQ specifically, in a study of 800 young offenders, 38.2% demonstrated elevated scores on the CTQ MD scale, indicating significant underreporting of abuse and neglect in this particular population [37]. Outside of forensic settings, is the MD scale a valid marker of a consequential response bias? On one hand, some researchers have suggested that when a patient has no external motivation to deceive the examiner, certain response biases may be either inconsequential, or even indicative of good mental health ([38], and see [39] for another perspective on “minimization” of early maltreatment). On the other hand, the denial of traumatic events in childhood can be associated with severe mental disturbance [40, 41]. To whit, even if the MD scale does reliably indicate a response bias, the impact or import of this bias on the CTQ scale’s validity is unclear.

The goals of the present study, then, were to answer three fundamental questions about minimization and its measurement with the MD scale. The first concerns its prevalence, asking: how common is it? The second concerns the MD scale’s characteristics, asking: Is MD characterized by types or degrees of response bias (i.e., is the latent construct discrete or continuous)? More pointedly, this second question concerns whether CTQ responses should be considered either valid or invalid based on a categorical interpretation of the MD subscale, or whether response bias is increasingly prevalent to the degree that MD scores are higher. The third and perhaps most important question concerns the consequence of minimization, asking: Does minimization moderate the discriminative validity of the CTQ in predicting a real-world outcome of interest (i.e. psychiatric illness)? Specifically, this final issue hinges on the well-established fact that childhood maltreatment is predictive of both internalizing and externalizing psychiatric disorders, is associated with an almost half of childhood-onset disorders and nearly a third of later-onset disorders, and approximately doubles the likelihood of a broad range of adverse mental health outcomes [1, 42–44]. Therewith, if response bias (here, MD positivity) indicates denial and the underreporting of maltreatment, and if this bias is consequential, the MD scale should moderate the discriminative validity of the CTQ, diminishing its ability to differentiate between psychiatric patients and community volunteers.

## Methods

### Participants

For this archival research study, a literature review was performed in peer-reviewed journals, recruiting research groups who had used the 28-item CTQ. Corresponding authors were contacted and asked to participate if their studies A) included the 28-item CTQ; and B) had a generous sample size (typically, at least 100 participants). Because our goal was to gather a large and generalizable sample, no further restrictions were placed on study inclusion. In all, de-identified, item-level data were collected from 24 samples provided by 21 researchers for a total of 19,652 participants. The studies included (see S1 Table) were conducted in Germany, the Netherlands, Norway, South Africa, South Korea, Sweden, Switzerland, Turkey, the United Kingdom, and the United States of America. In all, 7 different languages (and 7 different, validated versions of the CTQ [31, 33, 45–49]) were represented: English (*n* = 8,636), German (*n* = 7,557), Turkish (*n* = 1301), Swedish (*n* = 1,026), Dutch (*n* = 488), Norwegian (*n* = 481), and Korean (*n* = 163). The mean age of participants was 38 (*SD* = 16); 63% (*n* = 12,037) were female. Complete data on race and ethnicity were not available on all participants. This study used information that was recorded by the investigators in such a manner that subjects could not be identified, directly or through identifiers linked to the subjects, and therefore was certified as exempt by the Human Research Protection Program of the University of California, San Diego School of Medicine. Specifically, patient records and information was anonymized and de-identified prior to analysis.

Thirty-one percent of participants (*n* = 6,131) were psychiatric patients and the remaining 69% (*n* = 13,521) were community-based individuals not actively seeking psychiatric treatment. As data were combined from multiple, independent studies with different screening procedures and instruments, not all participants were systematically screened for all DSM or ICD psychiatric disorders (see S1 Table).

### Measures

#### Childhood Trauma Questionnaire

As previously described, the CTQ is a 28-item self-report inventory with five subscales (EA, PA, SA, EN, and PN) and one response bias subscale, the minimization and denial scale (MD) (Bernstein & Fink, 1998). Each subscale is composed of 5 items (except MD which is composed of 3) and require respondents to rate statements using 1 of 5 polytomous response options: (1) “never true”, (2) “rarely true”, (3) “sometimes true”, (4) “often true”, and (5) “very often true”. Two items from the PN subscale and five items from the EN subscale are reverse-coded. The CTQ manual [33] contains a table which classifies both subscale scores as well as CTQ total score into severity quintiles: “none/minimal” (EA < = 8, PA < = 7, SA = 5, EN < = 9, PN< = 7, CTQ < = 36), “low to moderate” (EA > 8 and < = 12, PA > 7 and < = 9, SA > 5 and < = 7, EN > 9 & < = 14, PN > 7 and < = 9, CTQ > 36 and < = 51), “moderate to severe” (EA > 12 and < = 15, PA > 9 and < = 12, SA > 7 and < = 12, EN > 15 and < = 17, PN > 9 and < = 12, CTQ > 51 and < = 68), and “severe to extreme” (EA > = 16, PA > = 13, SA > = 13, EN > = 18, PN > = 13, CTQ > = 69). Per the CTQ’s scoring instructions, MD item scores of 1 through 4 were coded as 0 and scores of 5 were coded as 1. “MD positivity,” then, means the MD score is greater than 0. The psychometric properties of the CTQ have been extensively validated in a number of English-speaking samples [33, 50, 51] and in every language in the current study, including: German [45]; Swedish [31]; Norwegian [46]; Turkish [49]; Korean [47] and Dutch [48]. In the current study, the reliabilities (α) were as follows: EA = .87; PA = .83; SA = .94; EN = .89; PN = .62; and MD = 0.68.

### Analyses

Besides documenting the frequency of minimization, our second goal was to determine whether the MD construct measured by the CTQ is best represented as a taxon (i.e., different types of minimization) or as a dimension (i.e., degrees of minimization). To do so, we relied on *taxometric analyses* [52–56], procedures that determine if relations among observed data are better accounted for by the presence of dimensional or categorical latent structure. We analyzed data using three separate taxometric procedures (mean above minus below a cut, MAMBAC; maximum eigenvalue, MAXEIG; and latent mode factor, L-Mode) with Ruscio’s (2012) taxometric program for R [57]. Inverted U-shaped graphs for the MAMBAC procedure, peaked graphs for the MAXEIG procedure, and multimodal distributions of factor scores for the L-Mode procedure are all suggestive of taxonic structure. Inverted U-shaped graphs for the MAMBAC procedure, peaked graphs for the MAXEIG procedure, and bimodal distributions of factor scores for the L-Mode procedure are all suggestive of taxonic structure. As part of the software used, taxonicity was judged based on parallel analyses of categorical and dimensional comparison data (see [58]). Specifically, the approach compares MAMBAC, MAXEIG, and L-Mode curves based on the observed data to curves based on categorical and dimensional simulations. The curves are plotted against the simulated data for comparison. Additionally, the results are summarized using the comparison curve fit index (CCFI; [55]). CCFI values range from 0 to 1; values closer to 0 indicate dimensional structure and values closer to 1 indicate categorical structure. If the taxometric results suggest dimensional structure, MD should be treated continuously, with higher scores indicative of increasing levels of minimization and denial; if the taxometric results suggest categorical structure, MD should be treated discretely, with scores used to determine either absence or presence of minimization, with no middle option.

To address our third goal—determining whether the MD subscale scores impact the discriminative validity of the CTQ for a meaningful, real-world variable [59]—we examined whether the MD scale moderated the relationships between CTQ total scores (or subscale scores) and patient versus community status, using a multilevel generalized linear model (see [60]) allowing a random intercept effect for language (*n*_*L*_ = 7). Fixed effects included gender, age, standardized MD total scores, and standardized CTQ total scores. We also included an interaction/moderation term for CTQ by MD. Data were analyzed using the lme4 package for *R* [61]. A logistic link with a binomial error distribution was used. In addition to standard output, we computed coefficients scaled in log-odds [exp(*b*)] and partial correlation coefficients (ρ_XY.Z_) for each effect.

## Results

**Fig 1:**
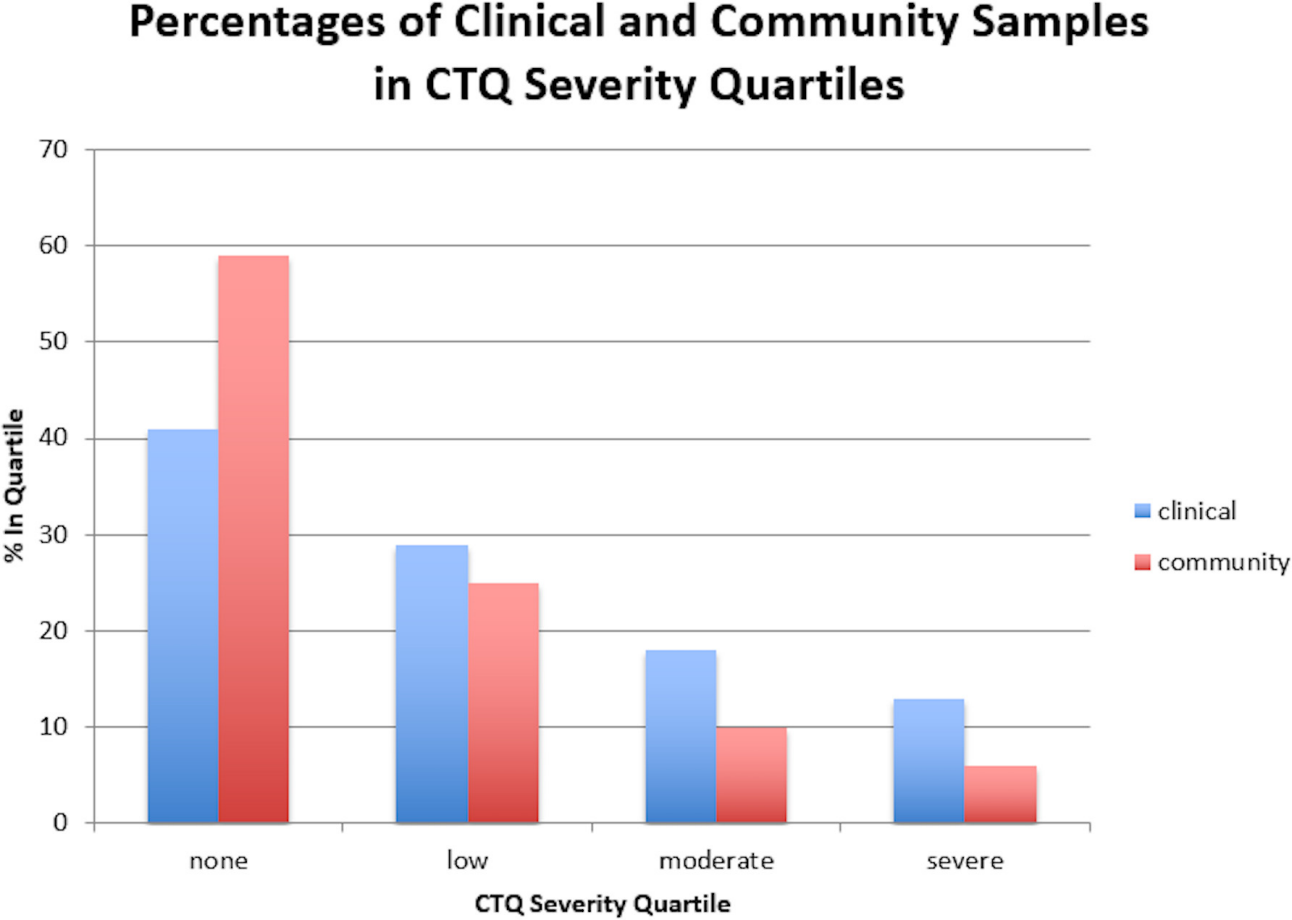
Percentages of Clinical and Community Samples in CTQ Severity Quartiles. X-Axis: Quartiles of childhood maltreatment based on total CTQ scores: none, low, moderate, and severe. Y-Axis: The percentage of subjects whose CTQ scores fall into that severity quartile. Within each quartile, the bar depicted on the left represents the percentage of clinical subjects (n = 5429–5876), and the bar on the right represents the percentage of community subjects (n = 12432–12915). Notably, the largest relative percentage of community subjects was in the “none” maltreatment quartile. That trend was reversed in the “moderate” and “severe” categories, where double the percentage of subjects were in the clinical group.

The average CTQ total score was 40.95 (*SD* = 15.56) across all samples, 38.78 (*SD* = 14.98) in community samples, and 45.91 (*SD* = 18.79) in patient samples. Means are reported in Table 1, and descriptive associations between CTQ severity ratings and the clinical versus community criterion variable are reported in Table 2. Table 2 also reports correlations between CTQ scores and the community versus clinical criterion. Patients consistently reported more childhood maltreatment compared to community participants (Fig 1); correlations representing these effects were in the small to medium range. As such, being in the clinical group was positively associated with CTQ total scores (*r*_pb_ = .20; *p* < .001). Fig 1 illustrates the relative percentages of clinical versus community patients in each severity quartile of childhood maltreatment.

**Table 1:** CTQ and subscale means for clinical and community samples.

	*EA*	*PA*	*SA*	*EN*	*PN*	*CTQ*	*MD*
Community (n = 12432–12915)*	8.14 (4.32)	7.06 (3.48)	6.64 (3.97)	9.80 (4.86)	7.32 (3.04)	38.78 (14.98)	0.74 (1.00)
Clinical (n = 5429–5876)*	10.35 (5.59)	7.55 (4.23)	7.17 (4.74)	12.87 (5.77)	8.42 (3.59)	45.91 (18.79)	0.46 (0.83)

*Total sample size is less than 19,652, and varies due missing data and listwise deletion.

**Table 2:** Associations between CTQ Total and Subscale Score Severity Ratings and Community (n = 12432–12915)* versus Clinical (n = 5429–5876)* Criterion.

	Sample	None	Low	Moderate	Severe	*r*_*pb*_
EA	Community	8745 (68%)	2190 (17%)	838 (7%)	1057 (8%)	0.21**
EA	Clinical	2931 (50%)	1192 (20%)	588 (10%)	1111 (19%)	0.21**
PA	Community	9656 (75%)	1432 (11%)	807 (6%)	1020 (8%)	0.06**
PA	Clinical	4163 (71%)	551 (9%)	457 (8%)	705 (12%)	0.06**
SA	Community	9677 (76%)	910 (7%)	1067 (8%)	1160 (9%)	0.06**
SA	Clinical	4101 (71%)	495 (9%)	557 (10%)	661 (11%)	0.06**
EN	Community	7307 (57%)	3348 (26%)	1027 (8%)	1132 (9%)	0.27**
EN	Clinical	2017 (35%)	1623 (28%)	766 (13%)	1391 (24%)	0.27**
PN	Community	8404 (65%)	2030 (16%)	1377 (11%)	1029 (8%)	0.16**
PN	Clinical	2870 (50%)	1129 (20%)	949 (17%)	782 (14%)	0.16**
CTQ	Community	7376 (59%)	3065 (25%)	1256 (10%)	726 (6%)	0.20**
	Clinical	2214 (41%)	1566 (29%)	959 (18%)	690 (13%)	

Note: Listwise deleted point biserial correlations (*r*_*pb*_) are reported for the associations between the dichotomous grouping variable (community = 0; clinical = 1) and continuous CTQ scale scores. Severity ratings based on CTQ manual. EA = Emotional Abuse subscale score; PA = Physical Abuse subscale score; SA = Sexual Abuse subscale score; EN = Emotional Neglect subscale score; PN = Physical Neglect subscale score; CTQ = Childhood Trauma Questionnaire total score.

*Total sample size is less than 19,652, and varies due missing data and listwise deletion.

** p < 0.001.

The average MD scale score was 0.66 (*SD* = 0.96) across all samples, 0.46 (*SD* = 0.83) in patient samples, and 0.74 (*SD* = 1.00) in community samples (Table 1). 42% of community samples were MD positive, versus 28% of clinical samples, and clinical samples scored significantly lower on the MD scale (*r*_pb_ = -.14, *p* < .001) (Table 3). MD scores demonstrated a strong negative correlation with CTQ total scores (-0.53; *p* < 0.001) (Table 4).

**Table 3:** Associations between MD Subscale Scores and Community versus Clinical Criterion.

	MD Items Endorsed “Very Often True”			
	0	1	2	3
Community	7055 (58%)	2373 (19%)	1717 (14%)	1083 (9%)
Clinical	3677 (72%)	751 (15%)	456 (9%)	224 (4%)
*r*_*rho*_	-0.14*			

Note: Listwise deleted Spearman correlations (*r*_*rho*_) are reported for the associations between the dichotomous grouping variable (community = 0; clinical = 1) and ordered categorical MD scale scores. MD = Minimization-Denial subscale total score. Total sample size is less than 19,652 due missing data and listwise deletion.

* p < 0.001.

**Table 4:** Association between CTQ Severity Ratings and MD Total Scores.

	MD Items Endorsed “Very Often True”			
CTQ	0	1	2	3
None	3885 (38%)	2050 (69%)	1854 (89%)	1227 (96%)
Low	3481 (34%)	551 (19%)	196 (9%)	33 (3%)
Moderate	1738 (17%)	198 (7%)	32 (2%)	13 (1%)
Severe	1040 (10%)	166 (6%)	10 (0%)	2 (0%)
*r*_*rho*_	-0.53*			

Note: Listwise deleted Spearman correlations (r_rho_) are reported for the associations between the dichotomous grouping variable (community = 0; clinical = 1) and continuous CTQ total scores. MD = Minimization-Denial subscale total score; CTQ = Childhood Trauma Questionnaire total score. Total sample size is less than 19,652 due missing data and listwise deletion.

* p < 0.001.

**Fig 2:**
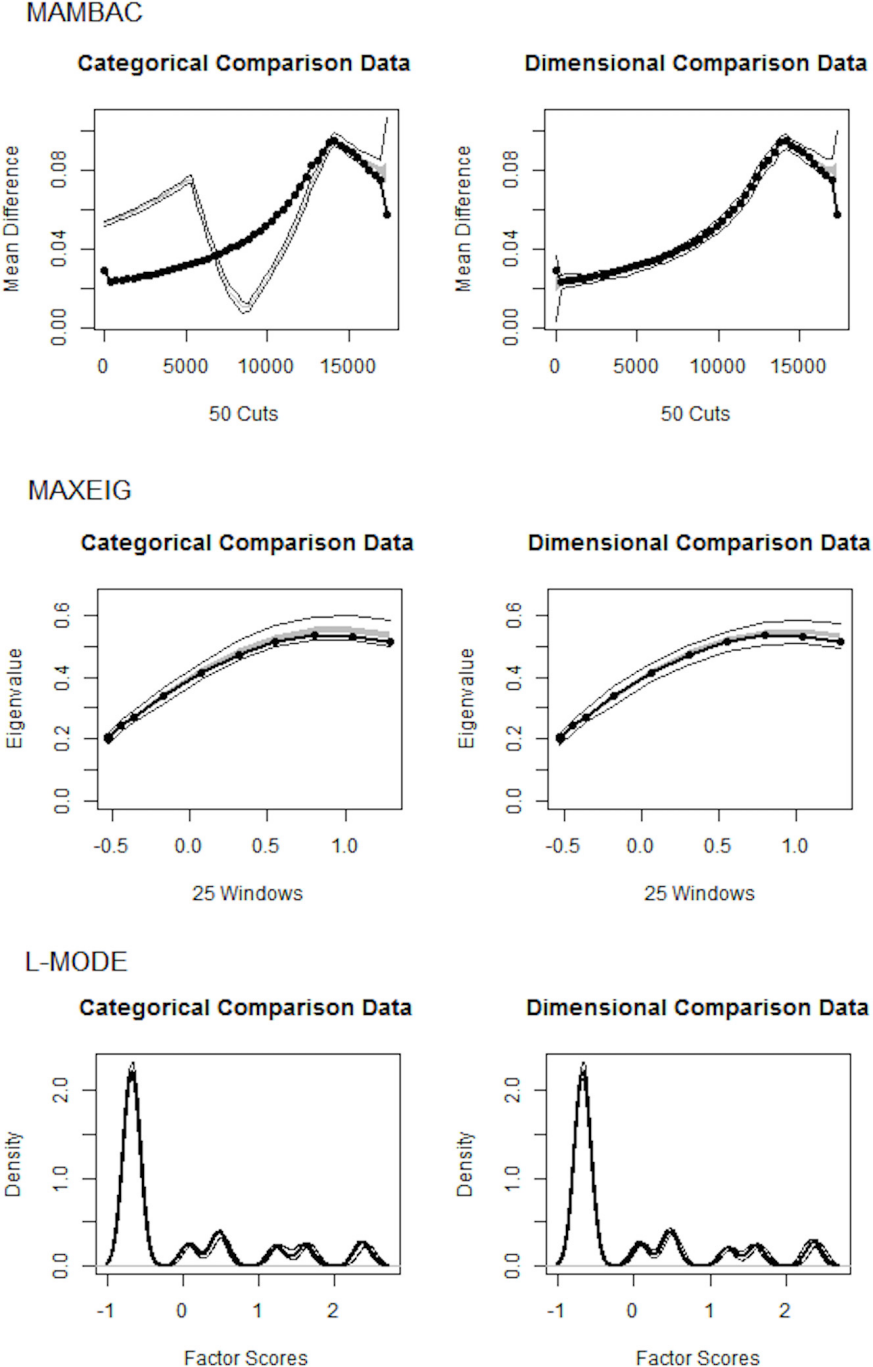
Taxometric Analyses of Minimization and Denial Items. Top row: left panel—average MAMBAC curve for the observed data (dark line) in comparison to simulated taxonic data (light lines representing one standard deviation above and below the mean); right panel—average MAMBAC curve for the observed data (dark line) in comparison to simulated dimensional data (light lines representing one standard deviation above and below the mean). Middle row: left panel—average MAXEIG curve for the observed data (dark line) in comparison to simulated taxonic data (light lines representing one standard deviation above and below the mean); right panel—average MAXEIG curve for the observed data (dark line) in comparison to simulated dimensional data (light lines representing one standard deviation above and below the mean). Bottom row: left panel—average L-Mode curve for the observed data (dark line) in comparison to simulated taxonic data (light lines representing one standard deviation above and below the mean); right panel—average L-Mode curve for the observed data (dark line) in comparison to simulated dimensional data (light lines representing one standard deviation above and below the mean). Inverted U-shaped graphs for the MAMBAC procedure, peaked graphs for the MAXEIG procedure, and bimodal distributions of factor scores for the L-Mode procedure are all suggestive of taxonic structure.

Fig 2 presents averaged curves, along with categorical and dimensional comparisons, for all three taxometric procedures. As can be seen, the averaged MAMBAC curve is highly consistent with the dimensional comparison data. Moreover the MAMBAC CCFI value of .12 supports a dimensional MD construct. The MAXEIG curve is more ambiguous, as is the MAXEIG CCFI value of .45, and only weakly favors dimensional structure. Although the L-Mode plot is somewhat multimodal, it is difficult to determine whether the curve is more consistent with either the categorical or dimensional comparison data. The L-Mode CCFI value of .31 suggests the latter.

Given that the overall taxometric results suggest that MD is consistent with a dimensional rather than categorical construct, a multilevel model was fitted to the data assuming a continuous MD variable (i.e., subscale total scores). The overall model’s pseudo *R*^2^ was .23. Without the main effect of MD, or the interaction between CTQ and MD, the pseudo *R*^2^ was .14. The main effects of gender, age, CTQ total scores, and MD subscale scores were all significant. Patients were more likely to be younger (*b* = -0.02 [CI_95%_ = -0.02, -0.01], *SE* = 0.001, *p* < 0.01, exp(*b*) = 0.98, ρ_XY.Z_ = 0.10), male (*b* = 0.19 [CI_95%_ = 0.10, 0.28], *SE* = 0.05, *p* < 0.01, exp(*b*) = 1.21, ρ_XY.Z_ = 0.03), have higher CTQ total scores (*b* = 0.35 [CI_95%_ = 0.30, 0.41], *SE* = 0.03, *p* < 0.001, exp(*b*) = 1.43, ρ_XY.Z_ = 0.11), and lower MD total scores (*b* = -0.09 [CI_95%_ = -0.16, -0.03], *SE* = 0.03, *p* < 0.01, exp(*b*) = 0.91, ρ_XY.Z_ = 0.02). The interaction between CTQ and MD total scores was also significant. CTQ total scores were less accurate in predicting patient status when MD subscale scores were high (*b* = -0.08 [CI_95%_ = -0.15, -0.01], *SE* = 0.04, *p* = 0.03, exp(*b*) = 0.92, ρ_XY.Z_ = 0.01). We next examined whether MD moderated associations between CTQ subscale scores and the clinical versus community criterion variable. Although the main effects for all subscale scores on the criterion were significant and negative (i.e., patients reported more abuse and neglect), the only significant interaction term was between EN and MD. As with CTQ scores, EN subscale scores were less accurate in predicting patient status when MD subscale scores were high (*b* = -0.16 [CI_95%_ = -0.22, -0.10], *SE* = 0.03, *p* < .001, exp(*b*) = 0.85, ρ_XY.Z_ = 0.04). Overall, these results indicate that MD subscale scores have a small but significant moderating effect on the relation between CTQ total scores and the clinical versus community criterion variable, and that this moderation effect is particularly pronounced for the EN subscale.

## Discussion

In this analysis of the Childhood Trauma Questionnaire’s (CTQ) Minimization and Denial (MD) scale, we report three main findings. First—despite the fact that its import has been marginalized in the vast majority of studies that utilize the CTQ—in this large, multinational sample, minimization (defined as MD positivity—see Methods) is not rare, occurring in about thirty percent of the CTQ scales from clinical subjects and forty percent of the scales from community subjects. Secondly, results indicate that the latent MD construct is characterized by degrees rather than types of response bias. That is, people vary along a continuum between low and high levels of minimization. Third, our data indicate that the MD subscale is consequential. Specifically, the strength of association between the CTQ and the probability of being in the patient sample (if CTQ was high), or being in the community sample (if CTQ was low), was attenuated by MD scores. This latter result provides evidence—for the first time that we are aware—that MD scores moderate the discriminative validity of the CTQ for a meaningful clinical outcome measures. Importantly, then, given that minimization is both common and consequential, these findings call into question the current practice of ignoring the MD scale, and support the scale’s intended function: earmarking certain people’s CTQ results for further investigation, analyses, or exclusion.

Consistent with prior findings that childhood maltreatment is correlated with a wide range of mental disorders [1, 12, 62], CTQ total scores (especially the EN and EA subscales) significantly predicted patient versus community status. Simply put, participants who reported more childhood experiences of abuse and neglect were more likely to be psychiatric patients. Though causality cannot be inferred from a cross-sectional sample, this result is consistent with previous metanalytic research that addresses issues of causality [1], and indicates that retrospectively-assessed childhood trauma has a causal role in increasing the risk for a wide range of psychiatric illness, including psychotic illness, mood disorders, dissociative disorders, anxiety disorders, substance use disorders, and personality disorders [1, 2, 62, 63]. Numerically, comparing our results with Baker’s review of another large (*n* > 1400), heterogenous, combined sample of clinical and community CTQ scores demonstrates a striking similarity between mean scores on the two subscales which showed the largest differences between clinical and community samples in our study: EA (10.1 vs 7.8) and EN (12.5 vs 9.4) compared with Baker’s EA (11.4 vs 8.5) and EN (12.5 vs 9.7) [17]. Though subtypes of maltreatment co-occur more often than not [64], and though historically, the impact of psychological maltreatment has been perhaps underemphasized (but see [65]), our findings again emphasize the unique impact of this more occult and less-studied subtype of maltreatment [62, 66].

Regarding minimization, MD scores were negatively related to being in the patient sample (i.e., decreased log odds). As mentioned in the introduction, the dichotomized scoring of MD items is designed specifically to identify response bias. It is possible that polytomous scoring of MD items (i.e., Gerdner’s aforementioned Idealization of the Upbringing construct; see [31]) would have a stronger effect. We chose not to explore this option, but it represents a possible direction for new research. High MD scores also attenuated the otherwise strong associations between high CTQ total scores and being in the patient sample as well as low CTQ total scores and being in the community sample. Thus, it appears that low CTQ scores in the presence of high MD scores are more likely to result in false negative diagnoses/classifications, consistent with the original design and purpose of the MD scale [33]. Given that childhood maltreatment increases the risk of psychiatric illness, and that the MD score attenuates that relationship, the most straightforward interpretation of our results is that they support the validity of the MD scale in detecting minimization and denial of trauma. Interestingly, when we examined the impact of MD on CTQ subscale scores, we found the EN subscale was particularly sensitive to the impact of minimization and denial. Reasons for this may include content overlap (four EN and two MD items contain the word “family”, for example), as well as the reality that EN (along with EA) was one of the subscales of the CTQ most predictive of our criterion variable in the first place.

Pragmatically, the results of this study suggest that the inclusion of MD-positive respondents in published studies using the CTQ may lead to attenuated relations between CTQ total and subscale scores (especially EN) and the various outcomes reported. In other words, findings from studies that: 1) used the 25-item CTQ (which excludes the MD subscale); or 2) use the 28-item CTQ (but fail to exclude MD-positive participants from analyses) may actually represent a conservative estimate of the true association between childhood trauma and its sequelae: exclusion of MD-positive participants from such analyses could strengthen associations. That said, future research is needed to determine how to best handle participants with high MD scores.

Expanding on this latter point, although the current findings support the practice of removal of MD-positive participants due to potential reporting bias (for example [25]), at least three practical issues warrant consideration. First, we found evidence that MD is characterized by degrees rather than types of response bias, and therefore, there is no simple cutoff MD score for valid versus invalid responding. Second, removing MD positive cases may result in restriction of range problems (i.e. a reduced range of CTQ scores), and may attenuate statistical relations with outcome variables in research studies. It is possible that more comprehensive or multidimensional psychometric models [67] may be able to account for the attenuating impact of MD without throwing away data. Third, the actual degree of attenuation produced by MD appears to be small, and therefore, its effect is likely most noticeable when sample sizes are large, when there are a large number of MD positive scores, and when outcome criteria have moderate base rates and strong associations with CTQ scales. The absence of these sample characteristics likely explains why some previous studies have failed to find an effect of MD moderating the association between CTQ scores and clinical variables (e.g.,[32]).

We highlight five limitations to the findings presented here. First, although the large sample size was a strength, it is possible that combining distinct, multinational and multilingual samples created unknown biases in the results. Though each study included in the analysis used a valid and reliable translation of the scale (see references above), measurement bias (invariance) due to language or cultural differences among the samples is a possibility [68]. Second, participants in the community sample were not systematically screened for psychiatric disorders. As such, although the patient versus community criterion variable is meaningful in itself, it is not a pure indicator of psychiatric illness due to criterion-group contamination. To the extent that community participants had undiagnosed or unacknowledged psychiatric illness (which is likely [69]), our results may actually underestimate the moderating effect of MD on the CTQ’s discriminative validity. Third, the relatively small absolute degree of moderation we found may be influenced by our particular sample. Other, different samples (for example, with a higher percentage of very low or very high CTQ scores) may have demonstrated either higher or lower degrees of moderation. Fourth, although we recognize the importance of brevity in response bias measures, the taxometric findings in this study are limited by the relatively small number of MD items. Taxometric analysis, moreover, cannot confirm the validity of the MD construct, and an assessment of the MD scale’s validity was not our aim. Fifth and lastly is the issue of the reliability of the MD scale. Though most studies that report on the CTQ’s reliability do not mention the reliability of the MD scale [16, 47, 70–73], and though some researchers who have examined this issue report the MD scale has low test-retest reliability (Daeho Kim, and Linde Martin, personal communication), others find it has satisfactory internal consistency (Arne Gerdner, personal communication). In this particular sample, the MD scale was—in point of fact—more reliable than the PN scale (see Methods), whose factor structure has repeatedly been questioned [31, 73–75]. Replication studies investigating response bias with a scale that contains a greater number of items are recommended.

In conclusion, our results call into question the widespread desuetude of the CTQ’s MD scale, and suggest that this frequently-ignored response bias scale does have a small but significant moderating effect on the CTQ’s discriminative validity. Clinical researchers and practitioners using the CTQ to study the prevalence or correlates of childhood maltreatment are advised to carefully identify study participants and patients with positive MD scores—particularly in the presence of very low CTQ scores—and consider whether their response data can be considered valid. Finally, to the extent that our findings are true, many of the reported effects of childhood maltreatment assayed by the CTQ may actually more significant than reported.

## Supporting Information

S1 Dataset This Excel spreadsheet that contains the pooled, raw, data from all of the collaborating investigators. This has been submitted at the request of the publishing entity, so that other researchers may also have access to the dataset used in our analyses.
(CSV)

S1 Table Samples included in the analysis. This table lists all of the data sets used for this research by primary investigator, providing the: number of community members in their sample; number of clinical patients used in their sample (alongside the type of clinical sample used); the language used by that research group; and a reference to where else these results were published. Validation studies for the foreign-language CTQ: German: (Wingenfeld, et al., 2010), Swedish:(Gerdner & Allgulander, 2009), Norwegian: (Dovran, et al., 2013) Turkish: (Sar, et al., 2012), Korean: (Kim, et al., 2011) Dutch: (Thombs, et al., 2009)
(DOCX)

## Abbreviations

Childhood Trauma Questionnaire (CTQ): a self-report questionnaire used to comprehensively evaluate the levels of various types of trauma experienced during childhood
Emotional Abuse (EA): subscale of the CTQ that evaluates the level of emotional abuse experienced during childhood
Emotional Neglect (EN): subscale of the CTQ that evaluates the level of emotional neglect experienced during childhood
Minimization Denial (MD): subscale of the CTQ that evaluates the level to which a person minimizes or denies trauma they may have experienced during childhood
Physical Abuse (PA): subscale of the CTQ that evaluates the level of physical abuse experienced during childhood
Physical Neglect (PN): subscale of the CTQ that evaluates the level of physical neglect experienced during childhood
Sexual Abuse (SA): subscale of the CTQ that evaluates the level of sexual abuse experienced during childhood
