# Supplementary material for: Minimization of Childhood Maltreatment Is Common and Consequential: Results from a Large, Multinational Sample Using the Childhood Trauma Questionnaire
Source: PLoS One. 2016 Jan 27;11(1):e0146058. doi: 10.1371/journal.pone.0146058 (PMC4729672; doi:10.1371/journal.pone.0146058)
Supplement: S1 Table — This table lists all of the data sets used for this research by primary investigator, providing the: number of community members in their sample; number of clinical patients used in their sample (alongside the type of clinical sample used); the language used by that research group; and a reference to where else these results were published. Validation studies for the foreign-language CTQ: German: (Wingenfeld, et al., 2010), Swedish:(Gerdner & Allgulander, 2009), Norwegian: (Dovran, et al., 2013) Turkish: (Sar, et al., 2012), Korean: (Kim, et al., 2011) Dutch: (Thombs, et al., 2009) (DOCX) [file pone.0146058.s002.docx]

**S1 Table. Samples included in the analysis.**

| **PI for Sample** | **Community** | **Clinical (sample type)** | **Language** | **reference:** |
| --- | --- | --- | --- | --- |
| Bohus | 0 | 443 (Borderline personality disorder) | German | ([Philipsen et al., 2008](#_ENREF_59)) |
| Bradley | 6281 | 0 | English | ([B. Bradley, Westen, et al., 2011](#_ENREF_12); [R. G. Bradley et al., 2008](#_ENREF_13)) |
| Dannlowski | 198 | 92 (Major depression, current episode, hospitalized patients) | German | ([Dannlowski et al., 2013](#_ENREF_17); [Dannlowski, et al., 2012](#_ENREF_18)) |
| Dorahy | 0 | 65 (DID patients , Schizophrenia patients) | English | ([Dorahy et al., 2009](#_ENREF_19)) |
| Fahlke | 0 | 367 (DSM-IV diagnosed alcohol dependence from an addiction treatment center) | Swedish | ([K. Berglund, Berggren, Fahlke, & Balldin, 2008](#_ENREF_5)) |
| Finzi-Dottan & Karu | 196 | 0 | Hebrew | ([Finzi-Dottan & Karu, 2006](#_ENREF_22)) |
| Gerdner | 239 | 420 (Anxiety disorder, substance use disorder, incest victims) | Swedish | ([Gerdner & Allgulander, 2009](#_ENREF_24)) |
| Glaesmer | 2504 | 0 | German | ([Hauser, Schmutzer, Brahler, & Glaesmer, 2011](#_ENREF_31); [Iffland, Brahler, Neuner, Hauser, & Glaesmer, 2013](#_ENREF_36); [Klinitzke, et al., 2012](#_ENREF_43)) |
| Grabe | 15 | 679 | German | ([Grabe et al., 2012](#_ENREF_26); [Grabe et al., 2010](#_ENREF_27)) |
| Kim | 0 | 163 (non-psychotic psychiatric outpatients) | Korean | ([Kim, et al., 2011](#_ENREF_41)) |
| Lobbestael | 226 | 262 (chronic fatigue syndrome patients ([Heins et al., 2011](#_ENREF_34)) ), | Dutch | ([Lobbestael, Arntz, Harkema-Schouten, & Bernstein, 2009](#_ENREF_47)) |
| Lochner | 0 | 408 (female psychiatric patients, patients with social anxiety disorder or panic disorder) | English | ([Lochner et al., 2002](#_ENREF_48)) |
| MacDonald | 0 | 200 (psychiatric outpatients) | English | ([MacDonald, et al., 2014](#_ENREF_49)) |
| Lauritzen and Ravndal | 0 | 481 (substance use disordered-patients) | Norwegian | ([Lauritzen, Ravndal, & Larsson, 2012](#_ENREF_44); [Ravndal, Lauritzen, Frank, Jansson, & Larsson, 2001](#_ENREF_61)) |
| Riggs | 286 | 0 | English | ([Riggs, Cusimano, & Benson, 2011](#_ENREF_63)) |
| Sar | 1301 | 0 | Turkish | ([Sar, Akyuz, Kugu, Ozturk, & Ertem-Vehid, 2006](#_ENREF_68); [Sar, Akyuz, Kundakci, Kiziltan, & Dogan, 2004](#_ENREF_69)) |
| Schäfer | 0 | 329 (patients with alcohol dependence) | German | Entire data set unpublished, though portion is from ([Schafer et al., 2007](#_ENREF_71)) |
| Schlosser | 0 | 821 (psychiatric patients) | German | Please refer to K Wingenfeld |
| Stein | 1335 (undergraduate students) | 61 (patients with anxiety disorders) | English | Has not yet been published |
| Subic-Wrana | 0 | 1439 (outpatients of the psychosomatic department of a university hospital) | German | ([Subic-Wrana et al., 2011](#_ENREF_79)) |
| Vogel | 0 | 193 (opioid-maintained, heroin-dependent patients) | German | ([Vogel et al., 2011](#_ENREF_88)) |
| Wingenfeld | 526 | 318 (psychiatric patients) | German | ([Wingenfeld, et al., 2010](#_ENREF_91)) |
